# Push–Pull Carbazole-Based Dyes: Synthesis, Strong Ultrafast Nonlinear Optical Response, and Effective Photoinitiation for Multiphoton Lithography

**DOI:** 10.1021/acsaom.4c00241

**Published:** 2024-08-05

**Authors:** Michalis Stavrou, Gordon Zyla, Dimitra Ladika, Frederic Dumur, Maria Farsari, David Gray

**Affiliations:** † Foundation for Research and Technology-Hellas, Institute of Electronic Structure and Laser, Heraklion 70013, Greece; ‡ Aix Marseille Univ, CNRS, ICR, UMR 7273, Marseille F-13397, France

**Keywords:** carbazole-based dyes, photoinitiators, ultrafast nonlinear optical properties, optical Kerr effect, multiphoton lithography

## Abstract

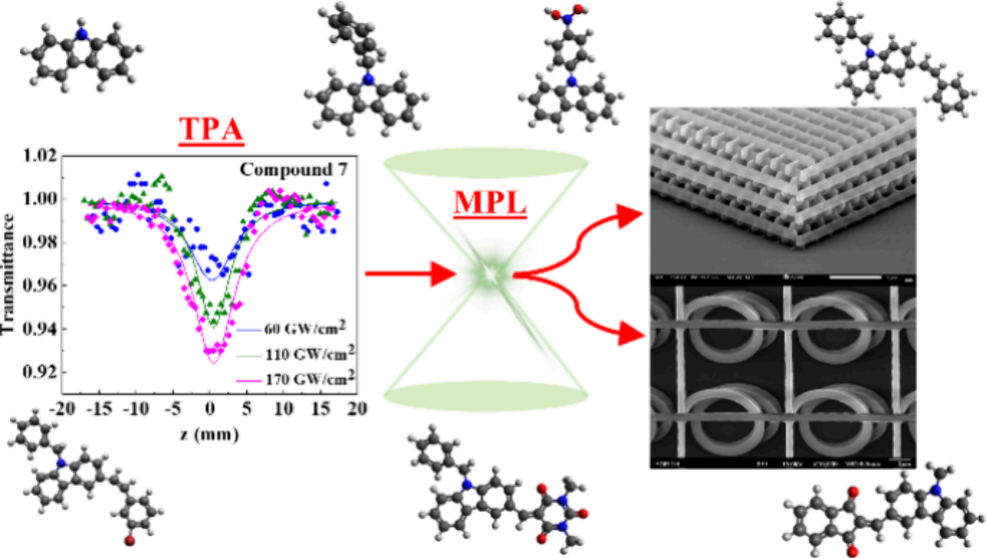

The present work reports on the ultrafast nonlinear optical (NLO) properties of a series of D−π–Α and D–A push–pull carbazole-based dyes and establishes a correlation between these properties and their efficiency for potential photonic and optoelectronic applications such as multiphoton lithography (MPL). The ultrafast NLO properties of the studied dyes are determined by two distinct experimental techniques, *Z*-scan and pump–probe optical Kerr effect (OKE), employing 246 fs laser pulses at 515 nm. The results indicate that chemical functionalization of the carbazole moiety with various strong electron-donating and/or electron-withdrawing groups, such as benzene, styrene, 4-bromostyrene, nitrobenzene, trimethyl isocyanurate, methyl, and indane-1,3-dione, can result in a controlled and significant enhancement of the NLO absorptive and refractive responses. In the context of potential applications, the efficiency of carbazole-based organic materials as photoinitiators (PIs) for MPL applications is demonstrated. The fabricated woodpile microstructure using chemically functionalized carbazole as a PI demonstrates improvements in both feature size and MPL efficiency compared to that using unfunctionalized carbazole as a PI. This is attributed to the efficient charge transfer resulting from chemical functionalization, which leads to a substantial increase (approximately 1 order of magnitude) in the values of the imaginary part of the second-order hyperpolarizability (Imγ) and the two-photon absorption cross section (σ). The achieved feature size of 280 nm is comparable to that obtained with other widely used PIs in MPL applications. Additionally, owing to the strong NLO properties of the studied functionalized carbazole, they could also be promising candidates for further applications in photonics and optoelectronics.

## Introduction

Over the past few decades, carbazole-based organic materials have garnered immense research interest owing to their extraordinary photophysical and electrical properties,^1−4^ diverse synthetic versatility,^5−8^ highly customizable spectroscopic characteristics,^9−11^ excellent thermal and photostability,^12,13^ and promising biological activities,^14^ among others. These characteristics endow carbazole-based dyes with a myriad of possibilities across diverse applications. From their integration into organic light-emitting diodes (OLEDs)^15−18^ and organic photovoltaics (OPVs)^19,20^ to their significant role in biomedical devices,^21^ chemical sensing,^22,23^ energy storage,^24,25^ and cationic photopolymerization,^26^ carbazole-based materials showcase promising possibilities in a wide range of technologies. In combination with their exceptional nonlinear optical (NLO) properties, including two-photon absorption (TPA),^27−29^ second harmonic generation (SHG),^30^ and optical Kerr effect (OKE),^31^ which are crucial in photonics and optoelectronics, carbazole derivatives hold promise as optical limiters, optical switchers, photonic integrated circuits, and photoinitiators (PIs) for additive manufacturing (AM), among others.

Until now, various versatile molecular architectures of π-conjugated carbazole dyes, such as those of the general type D–A, D−π–D, D−π–A, D−π–A−π–D, and A−π–D−π–A, where D and A denote electron-donating and -accepting groups attached on the carbazole moiety (π-conjugated backbone), have been reported.^32,33^ These architectures have demonstrated enhanced NLO response attributed to efficient charge transfer, achieved through controllable functionalization of carbazole moiety with strong D and A groups.^32,33^ Therefore, it is reasonable to expect that selecting D and A functional groups with lower and higher electronegativities, respectively, thereby creating a larger electron gradient within the molecule, can result in further enhanced optical nonlinearities, significantly enhancing the performance for specific applications. One such application that demands molecular systems with strong optical nonlinearities is multiphoton lithography (MPL). MPL stands out as an emerging manufacturing approach in high-resolution AM with unprecedented potential for future micro- and nanofabrication.^34−36^ In a standard MPL process, when a photosensitive material, consisting of reactive oligomers and a PI, is exposed to high-power radiation, the PI absorbs simultaneously two or more photons through virtual intermediate states, causing its decomposition into radicals. These radicals, in turn, induce a cross-linking reaction of the oligomers, leading to the solidification of the resin at the exposed regions. One of the main advantages of MPL is that it enables the localization of photochemically induced polymerization to the focal point of the incident laser beam, resulting in the precise fabrication of structures with feature sizes below the diffraction limit of light. Given these capabilities, MPL offers true 3D structuring at a very small scale, a superior manufacturing property over traditional lithographic approaches, proven to highly beneficial in various scenarios across multidisciplinary fields such as microelectronics,^37^ micro-optics/photonics,^38−40^ biomedicine,^41^ and microfluidics.^42^

In this context, the effectiveness of MPL, including resolution and fabrication time, is the primary effect of the effectiveness of the PI. In turn, the effectiveness of a PI can be characterized by its NLO absorption-related parameters, such as the imaginary part of the second-order hyperpolarizability (Imγ) and the two-photon absorption (TPA) cross section (σ).^43−48^ Among the reported effective PIs for initiating photopolymerization are benzophenone, benzylidenacetone/-cycloalkane, anthraquinone, fluorenone, thioxanthone, fluorene, anthracene, stilbene, triphenylamine, and phenothiazine derivatives.^49^ Thanks to their strong optical nonlinearities, traditional MPL-based 3D printing has successfully achieved high-resolution features of submicrometers.^50^ Moreover, more recent studies have demonstrated ongoing advancements in MPL technology, achieving feature sizes below 100 nm.^51−54^ However, many research groups that assess the effectiveness of PIs by determining TPA cross sections using high-repetition rate (as, e.g., MHz) femtosecond (fs) laser sources have led to the erroneous conclusion that a high cross section is not responsible for high efficiency.^55−58^ Primarily, under such experimental conditions, unwanted thermal effects inevitably manifest, obscuring pure electronic response contributions to the NLO response.^59^ As a result, erroneous extremely large values of the TPA cross section have been reported.

**Figure 1:**
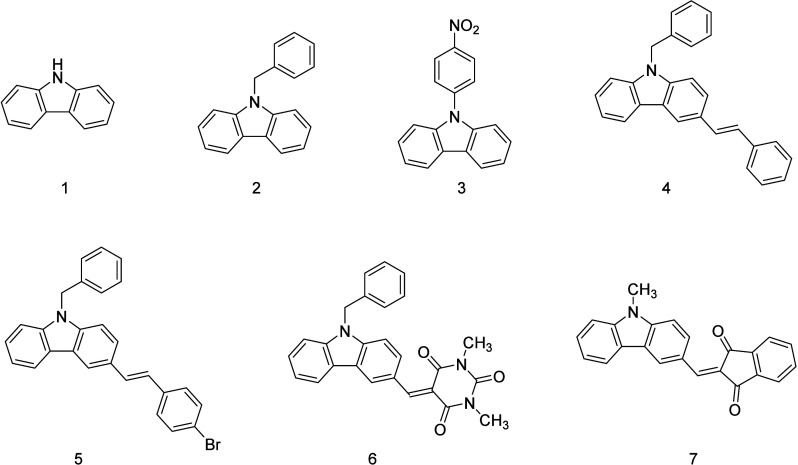
Chemical structures of the investigated carbazole-based dyes.

Carbazole could be also considered as the building block for synthesizing of efficient PIs because it includes multiple aspects for chemical functionalization, such as N atoms and aromatic rings. These aspects allow for linking with various functional groups acting as either electron donors or acceptors. Moreover, carbazole can serve as electron-donor units in D–A compounds. Therefore, the ability of carbazole to participate in charge transfer processes, whether as an electron-donating group or a π-bridge, can lead to enhanced optical nonlinearities, rendering carbazole-based materials very promising candidates for synthesizing efficient PIs. However, pertaining to their potential as PIs, only a few groups have exploited the charge transfer processes in carbazole-based dyes for initiating polymerization or cross-linking reactions,^60,61^ whereas studies on the influence of peripheral functionalization of carbazole on the MPL performance are rather scarce. In this context, this work systematically investigates the NLO properties and photoinitiator efficiency of a series of push–pull carbazole-based dyes. These dyes were functionalized with strong electron-donating and/or -accepting substituents such as benzene, styrene, 4-bromostyrene, nitrobenzene, trimethyl isocyanurate, methyl, and indane-1,3-dione (Figure 1). The results were compared with those obtained for unfunctionalized carbazole. All of the studied carbazole-based dyes were found to demonstrate a strong NLO response (i.e., second-order hyperpolarizabilities of the order of ∼10^–33^ esu) attributed to efficient charge transfer, as determined by using *Z*-scan and pump–probe optical Kerr effect (OKE) techniques. As a result, they show a high potential for fabrication of 3D microstructures with high resolution features, further positioning them as promising candidates for various applications in photonics and optoelectronics.

## Materials and Experimental Methods

### Compound Synthesis

All compounds were synthesized according to the procedures described in depth elsewhere.^62,26^

### Linear Characterization Methods

The UV–vis absorption properties of the compounds were investigated using a PerkinElmer Lambda 25 spectrophotometer, and their fluorescence activity was assessed employing a Horiba FluoroMax-3 fluorometer. For these spectroscopic measurements, 0.1 mg of powder of each compound was dissolved in 1 mL of dichloromethane (DCM). All of the spectra were taken using 1 mm thick glass cells.

### Computational Method

Density functional theory (DFT) calculations were carried out to optimize the molecular orbitals of each compound by employing the B3LYP method at the B3LYP/6-31G** level of theory. All the molecular orbital calculations were conducted with the Gaussian 09 package.^63^ The HOMO–LUMO gap values were computed from the energy difference between the HOMO and LUMO Kohn–Sham (KS) orbitals.

### Nonlinear Characterization Methods

**Figure 2:**
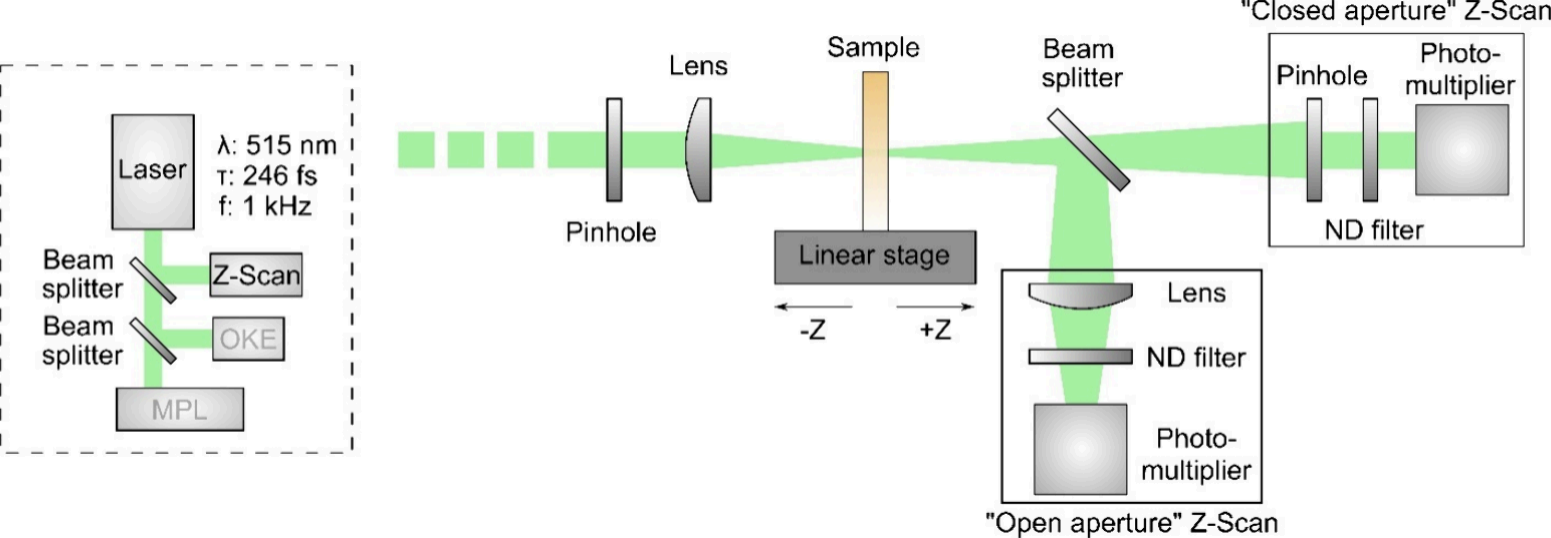
A schematic of the *Z*-scan experimental setup.

At first, the study of the NLO properties (NLO absorption and refraction) of the studied dyes was conducted utilizing the *Z*-scan technique.^64^ A schematic of the *Z*-scan experimental setup is shown in Figure 2. Briefly, in this technique, the normalized transmittance of a sample exposed to varying laser intensities as it traverses along the propagation direction (i.e., *z*-axis) of a focused laser beam is monitored by two distinct experimental configurations: the “open-aperture” (OA) and “closed-aperture” (CA) *Z*-scans. In the former configuration (i.e., the OA *Z*-scan), the entire laser beam passing through the sample is totally collected by a large-diameter lens and then measured by a photodetector, such as a photomultiplier. Simultaneously, in the latter configuration (i.e., the CA *Z*-scan), only a fraction of the transmitted beam is measured after passing through a narrow pinhole placed in the far field, just before a second photodetector. The obtained OA and CA *Z*-scan recordings enable the determination of the nonlinear absorption coefficient β and the nonlinear refractive index *n*_2_, respectively.

Typically, the OA *Z*-scan measurements can reveal either a transmission minimum or maximum at the focal plane, denoting reverse saturable absorption (RSA, β > 0) or saturable absorption (SA, β < 0), respectively. Accordingly, the OA *Z*-scan measurements can display a prefocal transmission minimum followed by a postfocal maximum or vice versa, indicating self-focusing (*n*_2_ > 0) or self-defocusing (*n*_2_ < 0) action, respectively. From the collected OA and CA *Z*-scan curves, the values of β and *n*_2_ can be determined through fitting with eqs 1 and 2, respectively:^64^
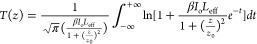
1
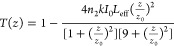
2where *T*(*z*) is the sample’s transmittance at each *z*-position, *z*_0_ is the Rayleigh length, *I*_0_ is the laser intensity at the focal plane, *L*_eff_ is the sample’s effective length, and *k* is the excitation wavenumber.

Subsequently, using the determined values of β and *n*_2_, the real and imaginary parts of the third-order susceptibility χ^(3)^ can be derived through eqs 3 and 4, respectively:
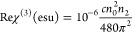
3
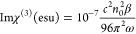
4where *c* is the speed of light, *n*_0_ is the linear refractive index, and ω is the laser frequency.

Given that χ^(3)^ is a macroscopic quantity affected by solute’s concentration, the second-order hyperpolarizability γ is frequently favored. This parameter, serving as a molecular constant, expresses the NLO response per molecule, thereby simplifying comparisons to other molecular systems. The real (Reγ) and imaginary (Imγ) parts of second-order hyperpolarizability γ can be calculated using the following relations:
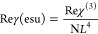
5
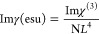
6where *N* represents the number of molecules/cm^3^ and *L* = (*n*_0_^2^ + 2)/3 is the local field correction factor.

Moreover, the obtained values of the nonlinear absorption coefficient β can be used to determine the TPA coefficient σ, employing the following relation:
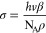
7where *h* is the Planck constant, *N*_A_ is the Avogadro number, and ρ is the concentration in mM.

**Figure 3:**
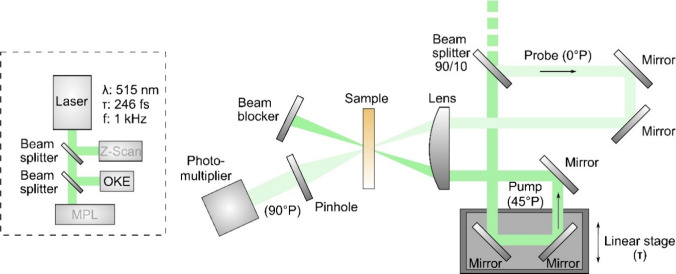
A schematic of the OKE experimental setup.

Then, the pump–probe optical Kerr effect (OKE) technique was utilized for determining the real part of second-order hyperpolarizability, offering insights into the dynamics of the induced NLO response and the mechanisms responsible for variation in the sample’s refractive index.^65^ A schematic of the OKE experimental setup is presented in Figure 3. This technique involves splitting the laser beam into two components, an intense pump beam and a weaker probe beam, with an intensity ratio of ∼9:1, using a 10/90 beam splitter. The pump beam is used to induce birefringence in the medium, which is then detected by the probe beam.

Both beams are initially linearly polarized with their polarization planes set to form a 45° difference. They traverse different optical paths through a Mach–Zehnder-type interferometer and spatially overlap into the sample by a 20 cm focal length quartz lens. When the time delay between the pump and probe beams is zero, the transmitted probe beam becomes elliptically polarized. Consequently, it can pass through a Glan–Thompson polarizer, with its optical axis perpendicular to the initial polarization of the probe beam. The signal recorded by a photomultiplier, referred to as the OKE signal, provides the value of Reχ^(3)^ by comparison with a reference material, using eq 8:^65^
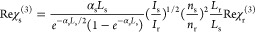
8

In this equation, subscripts s and r stand for the studied sample and the reference material, respectively; *I* is the OKE signal; *n* is the linear refractive index; α is the linear absorption coefficient; and *L* is the effective length.

To explore the temporal evolution of the sample’s NLO response and its underlying mechanisms, one of the arms of the interferometer is equipped with a translation stage. This stage introduces a temporal delay between the pump and probe beams, allowing for an investigation of how the NLO response changes over time.

For *Z*-scan and OKE experiments, the second-harmonic generation (SHG) output at 515 nm from a fiber laser (aeroPULSE FS10), emitting laser pulses with a duration of 246 fs, was used. The laser repetition rate was set at 1 kHz through external triggering with a pulse-delay generator (Berkeley Nucleonics) to prevent the manifestation of unwanted thermal effects. The laser beam was focused into a 1 mm path length glass cell containing 0.1 mg/mL solutions of the compounds in DCM by means of a 20 cm focal length quartz planoconvex lens. The beam radius, *w*_0_, at the focal plane was accurately measured by a CCD camera, yielding a value of about 16 ± 2 μm.

### Photopolymer Synthesis and Optical Setup for MPL

To demonstrate the applicability of push–pull carbazole-based dyes as PIs for MPL, the compounds 1 and 7 illustrated in Figure 1 were incorporated with a concentration 1% w/w with respect to the organic in a standard MPL-photopolymer, an organic–inorganic zirconium–silicon hybrid composite^66^ similar to SZ2080,^67^ which exhibits large photopolymerization thresholds.^68,69^ For the synthesis of the composite, all chemicals were sourced from Sigma-Aldrich and employed without further purification. More specifically, the composite contained methacryloxy-propyl trimethoxysilane (MAPTMS 97%), zirconium propoxide (ZPO, 70% in propanol), and (dimethylamino)ethyl methacrylate (DMAEMA > 99%), which fosters quenching.^50,70^ In this context, MAPTMS and DMAEMA acted as the organic photopolymerizable monomers, whereas ZPO and the alkoxysilane groups of MAPTMS facilitated the formation of the inorganic network. The molar ratios to form the organic and inorganic network were MAPTMS/ZPO = 8:2 and (MAPTMS + ZPO)/DMAEMA = 9:1.^66^ Initially, MAPTMS underwent hydrolysis with the addition of HCl (0.1 M) followed by stirring for 15 min. In a separate flask, ZPO was chelated by adding DMAEMA in the presence of 145 μL of 1-propanol (1-PrOH) and left to stir for 20 min. Subsequently, the hydrolyzed MAPTMS was added dropwise to the ZPO solution and stirred for an additional 20 min. Finally, the composite material was filtered by employing 0.22 μm pore size filters. Compounds 1 and 7 were utilized as PIs with a concentration 1% w/w to the final solution of MAPTMS and DMAEMA composite, corresponding to mole percentages of 1.949 and 0.984% for compounds 1 and 7, respectively. The samples were prepared by drop casting onto 130 μm thick coverslip and air-dried for 5 days before photopolymerization.

**Figure 4:**
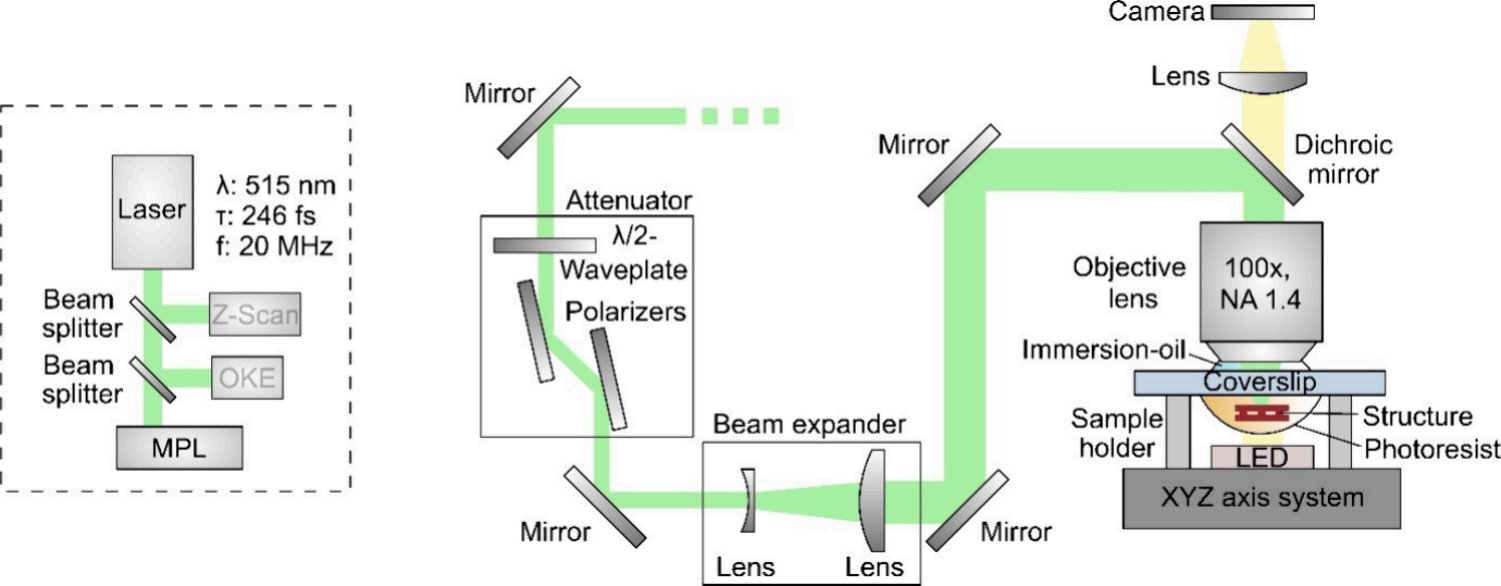
A schematic of the MPL experimental setup.

As proof of concept for efficient MPL involving push–pull carbazole-based dyes, the fabrication of photonic crystal-type structures, including woodpiles and spirals, was targeted to demonstrate the capability of high-resolution AM. To conduct MPL, a home-built experimental setup was employed with details of the optical setup presented in Figure 4.

A 100× microscope objective lens (Zeiss, Plan Apochromat, N.A. = 1.4 Oil DIC) was used to focus the laser beam into the photosensitive material. Specifically, for the realization of 3D structures, the sample was placed on a holder that was mounted on a high-precision XYZ piezoelectric axis system (Nanocube, Physik Instrumente). The axis system facilitates layer-by-layer manufacturing and allows for arbitrary translation of the sample relative to the focused laser beam.

The setup utilized the same laser source previously mentioned for the *Z*-scan and OKE approaches. However, for MPL, the laser’s repetition rate was set to 20 MHz. Additionally, the laser power could be precisely adjusted by using an attenuator (Altechna).

It is noteworthy that this is the first study in the literature where the investigation of the NLO properties of PIs and the manufacturing experiments has been conducted using the same laser source, thereby leading to more accurate conclusions for the effectiveness of PIs.

## Results and Discussion

**Figure 5:**
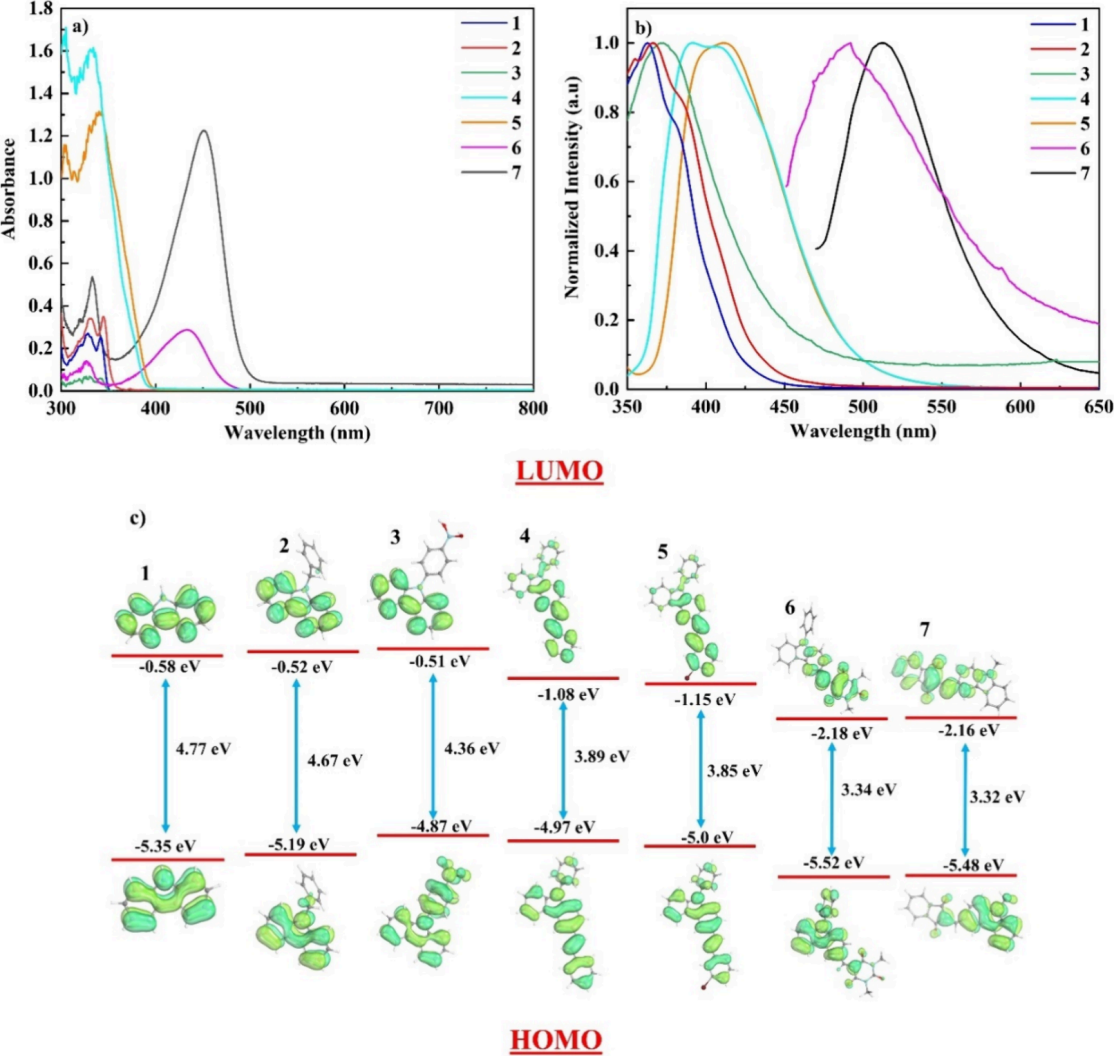
(a) UV–vis absorption spectra of compounds 1 (1.1 mM), 2 (0.78 mM), 3 (0.35 mM), 4 (0.56 mM), 5 (0.78 mM), 6 (0.24 mM), and 7 (0.35 mM) in DCM; (b) corresponding normalized emission spectra; and (c) HOMO and LUMO electronic structures calculated at the B3LYP/6-31G** level of theory.

The UV–vis absorption spectra of the solutions containing compounds 1 (1.1 mM), 2 (0.78 mM), 3 (0.35 mM), 4 (0.56 mM), 5 (0.78 mM), 6 (0.24 mM), and 7 (0.35 mM) in DCM are depicted in Figure 5a. As shown, the spectra of all the compounds show a characteristic absorption peak at around 300 nm, assigned to transitions from occupied π states to unoccupied π* states of the aromatic C=C bonds.^71^ In addition, an adjacent peak is observed in the range between 325 and 345 nm, originating from n-π* electronic transitions due to the presence of lone pairs (antibonding orbitals) on nitrogen atoms.^71^ Among all the carbazole-based dyes, compounds 6 and 7 present a broad absorption band in the visible region (i.e., above 400 nm), which can be described in terms of intramolecular charge transfer (ICT) from trimethyl isocyanurate and 9-methycarbazole donors to benzene and Indane-1,3-dione acceptor moieties, respectively. For all of the other compounds, there are less intense ICT-induced peaks located in the UV region, specifically between 340 and 360 nm. It should be highlighted that these peaks are less intense in the case of compounds 4 and 5, indicating the presence of ICT, as evidenced by the shoulders at approximately 340 and 360 nm.

In Figure 5b, the corresponding fluorescence spectra of carbazole-based dye solutions are illustrated. These compounds were found presenting their strongest fluorescence emission peak either in the ultraviolet or in the visible spectral region, with the main emission peaks shifting toward longer wavelengths from compound 1 to 7. This red shift phenomenon is likely due to increase of π-conjugation resulting from the attachment of electron-donating and -withdrawing groups. Compounds 3–7 exhibit a larger Stokes shift, ranging from 50 to 70 nm, compared to compounds 1 and 2, which demonstrate a Stokes shift of approximately 20 nm. This difference denotes that the excited state of their intramolecular charge has greater polarity and redistribution, thereby confirming their heightened molecular charge transfer ability.

The charge distributions of compounds 1–7 in both the ground and excited states, together with their corresponding molecular structures, were analyzed and optimized by DFT calculations at the B3LYP/6-31G** level of theory. In Figure 5c, the HOMO and LUMO orbitals as well as the orbital energies of the studied carbazole-based organic materials are depicted. From the inspection of the optimized geometries of the molecular orbitals, it becomes apparent that all the compounds demonstrate intramolecular charge transfer (ICT), with the charge transfer in compounds 3, 6, and 7 being stronger than that of all the other dyes. The energy band gap of carbazole (compound 1) decreases upon functionalization with different functional groups, in accordance with the red-shift observed in the UV–vis absorption spectra presented in Figure 5a. Among all the studied dyes, compounds 6 and 7 present the lowest values of energy band gap, indicating more extended π-electron delocalization, which enhances their charge transfer efficiency.

**Figure 6:**
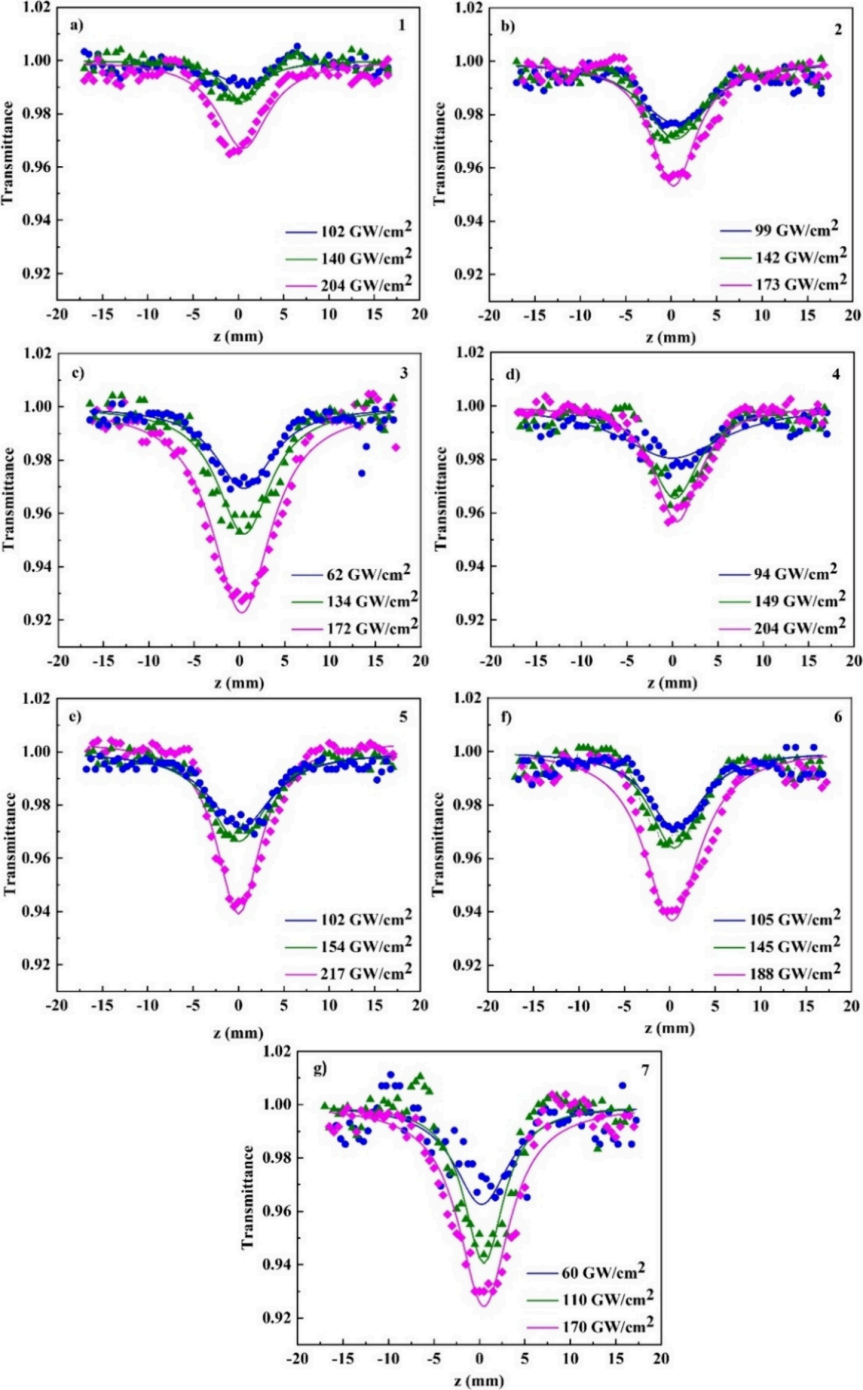
OA *Z*-scans for solutions of compounds (a) 1 (1.1 mM), (b) 2 (0.78 mM), (c) 3 (0.35 mM), (d) 4 (0.56 mM), (e) 5 (0.78 mM), (f) 6 (0.24 mM), and (g) 7 (0.35 mM) under different laser excitation intensities.

**Figure 7:**
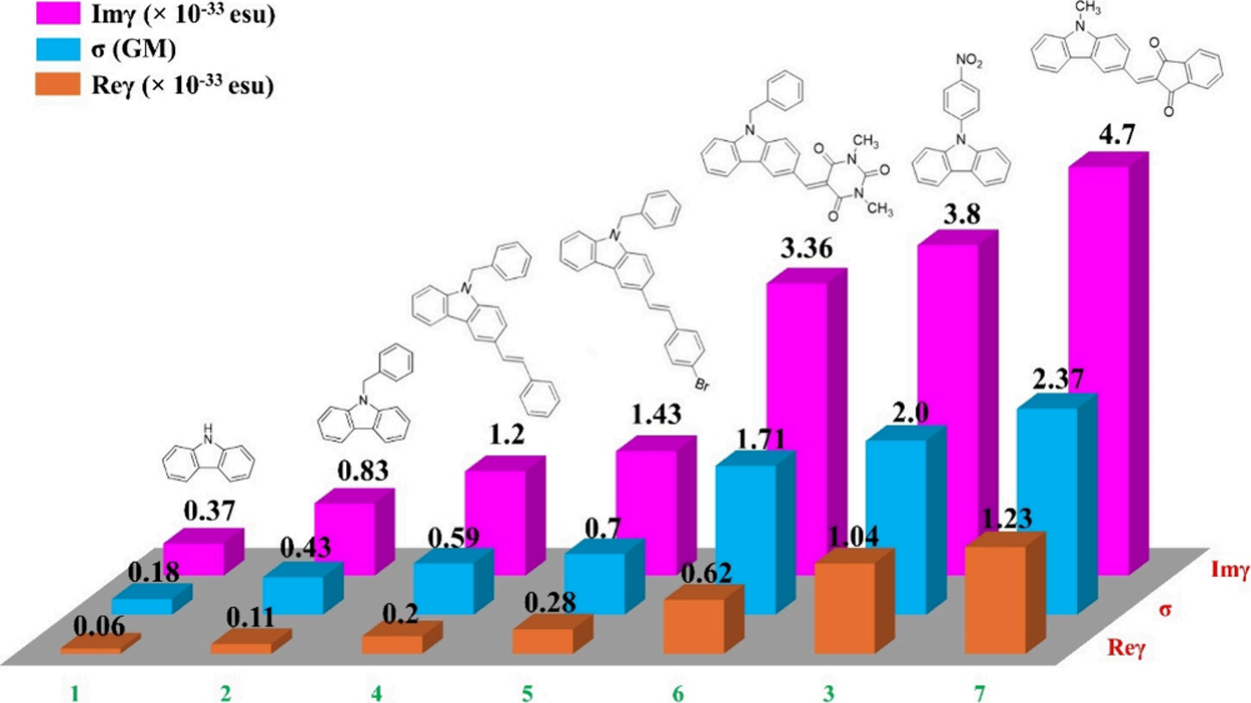
Magnitude of the imaginary part of second-order hyperpolarizability (Imγ), two-photon absorption cross section (σ), and real part of second-order hyperpolarizability (Reγ) of compounds 1–7 under 246 fs, 515 nm laser pulses.

In Figure 6, some representative OA *Z*-scans of compounds 1–7 are shown, acquired under 246 fs and 515 nm excitation, with varying laser intensities. The solid lines depicted in this figure correspond to the optimal fits for the experimental data points (indicated by solid symbols) using eq 1. Given DCM’s negligible NLO absorption within the laser intensity range (i.e., from 60 to 217 GW/cm^2^) employed, the shape of the OA *Z*-scan curves facilitates straightforward determination of the sign of nonlinear absorption coefficient β. From these recordings, it is clearly depicted that all the OA *Z*-scans reveal a minimum at the focal point (i.e., at *z* = 0), which increases with laser radiation intensity. This implies a positive absorptive nonlinearity, denoted by β > 0, indicative of a saturable absorption (RSA) behavior. Considering the lack of any absorption feature at the excitation wavelength (i.e., 515 nm) and the existence of electronic states lying in the UV spectral region (as evidenced by the UV–vis absorption spectra in Figure 5a), two- or multiphoton absorption processes can be regarded as potential mechanisms underlying the NLO absorptive response of the investigated molecules. By fitting the OA *Z*-scan recordings measured under various incident laser intensities to eq 1, the average values of β were determined. Subsequently, utilizing eqs 4, 6, and 7, the imaginary parts of third-order susceptibility (Imχ^(3)^) and second-order hyperpolarizability (Imγ), as well as the two-photon cross sections (σ), were calculated for all the carbazole-based dyes. The resulting values for each molecule at various solution concentrations are gathered in Table S1. It is noteworthy that among these NLO absorption-related parameters, only Imγ and σ remain unaffected by concentration, providing a figure of merit for the absorptive nonlinearity across the different concentration samples. Hence, for clarity, these concentration-independent parameters are schematically represented in Figure 7, facilitating comparisons. From a simple inspection of this figure, it is clearly indicated that the chemical functionalization of carbazole moiety with different electron-donating and/or -accepting substituents results in a substantial enhancement of its NLO absorption. The possible mechanisms underlying this enhancement are elucidated below. As shown in Table S2, the determined NLO absorption-related parameters of the present carbazole-based organic materials are comparable to those of other dyes reported as highly efficient radical initiators, such as 4,4′-bis(diethylamino)benzophenone,^43^ Irgacure series,^44^ thioxanthones,^45,46^ ketones,^47^ and acylophosphine oxide.^48^ As a result, the studied dyes are expected to be promising candidates for initiating photopolymerization in MPL applications.

**Figure 8:**
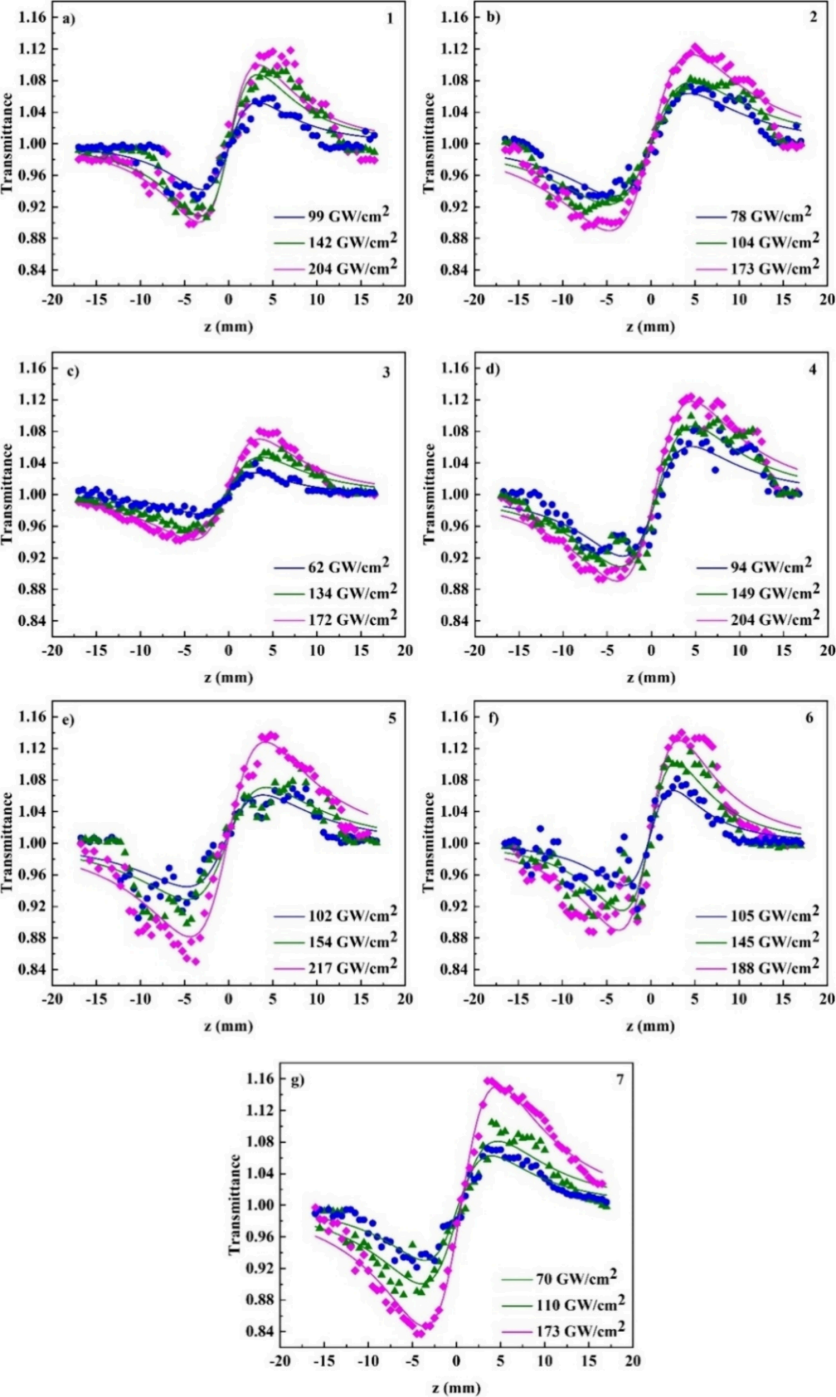
CA *Z*-scans for solutions of compounds (a) 1 (1.1 mM), (b) 2 (0.78 mM), (c) 3 (0.35 mM), (d) 4 (0.56 mM), (e) 5 (0.78 mM), (f) 6 (0.24 mM), and (g) 7 (0.35 mM) under different laser excitation intensities.

For the assessment of the NLO refractive response of compounds 1–7, CA *Z*-scan recordings were measured by employing various laser excitation intensities under 246 fs laser pulses at 515 nm. Typical characteristic CA *Z*-scans are shown in Figure 8, with the continuous lines corresponding to the best possible fit of the experimental recordings through eq 2. As demonstrated, all the solutions exhibit a valley–peak transmittance configuration, suggesting a self-focusing behavior (i.e., *n*_2_ > 0). It is important to note at this point that the neat solvent (i.e., DCM) was found to display measurable NLO refraction, particularly self-focusing within the range of incident laser light intensities used in the experiments (Figure S1). Consequently, the solvent’s influence on the values of *n*_2_, Reχ^(3)^, and Reγ has been subtracted using the typical procedures for analyzing *Z*-scan data.^64^ As indicated in Table S1, after the contribution of the solvent was removed, the values of NLO refraction-related parameters of compound 3 were found to be negative, denoting self-defocusing. Conversely, all other studied compounds displayed positive values, signifying self-focusing. Similarly to NLO absorption, the type of functional groups attached to the surface of carbazole led to controllable enhancement of NLO refraction, as evidenced from the values of the real part of second-order hyperpolarizability also included in Figure 7.

To delve into the mechanisms leading to the enhanced NLO response (NLO absorption and refraction) of the functionalized carbazole derivatives, it is essential to examine their electronic transitions and chemical structures. More precisely, in this study, two distinct molecular architectures of push–pull carbazole-based dyes, namely, D–A (compounds 2 and 3) and D−π–Α (compounds 4–7), were investigated. Comparing the values of Imγ, σ, and Reγ presented in Figure 7 for the samples belonging to the first category of molecules (compounds 2 and 3), it is observed that chemically functionalized carbazole with nitrobenzene demonstrates a considerably stronger NLO response than its benzene-functionalized counterpart. This heightened NLO response can be ascribed to efficient charge transfer from carbazole to styrene, induced upon transitions from HOMO to LUMO+1, as evident in Figure S2, because 515 nm laser irradiation, corresponding to a photon energy of ∼2.4 eV, can bridge the energy gap between LUMO and HOMO+1 states, which was found to be ∼4.7 eV, with two photons. On the other hand, in the case of compound 2, under 515 nm laser irradiation, probably leading to electronic transitions from LUMO to HOMO states through TPA, the extent of charge transfer appears to be relatively low as shown in Figure 5c and Figure S2. Regarding the second category of samples, namely, the D−π–Α molecules, compounds 4–7 feature benzene and indane-1,3-dione as electron acceptors connected to electron donors, such as styrene, bromostyrene, trimethyl isocyanurate, and methyl, through a conjugated spacer (i.e., carbazole). As can be seen in Figure 7, among these dyes, compound 7 exhibited the strongest NLO response followed by compounds 6, 5, and 4. This enhancement can be explained by the decreasing trend in the energy band gaps from compound 4 to 7 (Figure 5c), which is further supported by their red-shift in their UV–vis-NIR absorption spectra (Figure 5a), implying a more extended π-electron delocalization, facilitating more efficient charge transfer.

**Figure 9:**
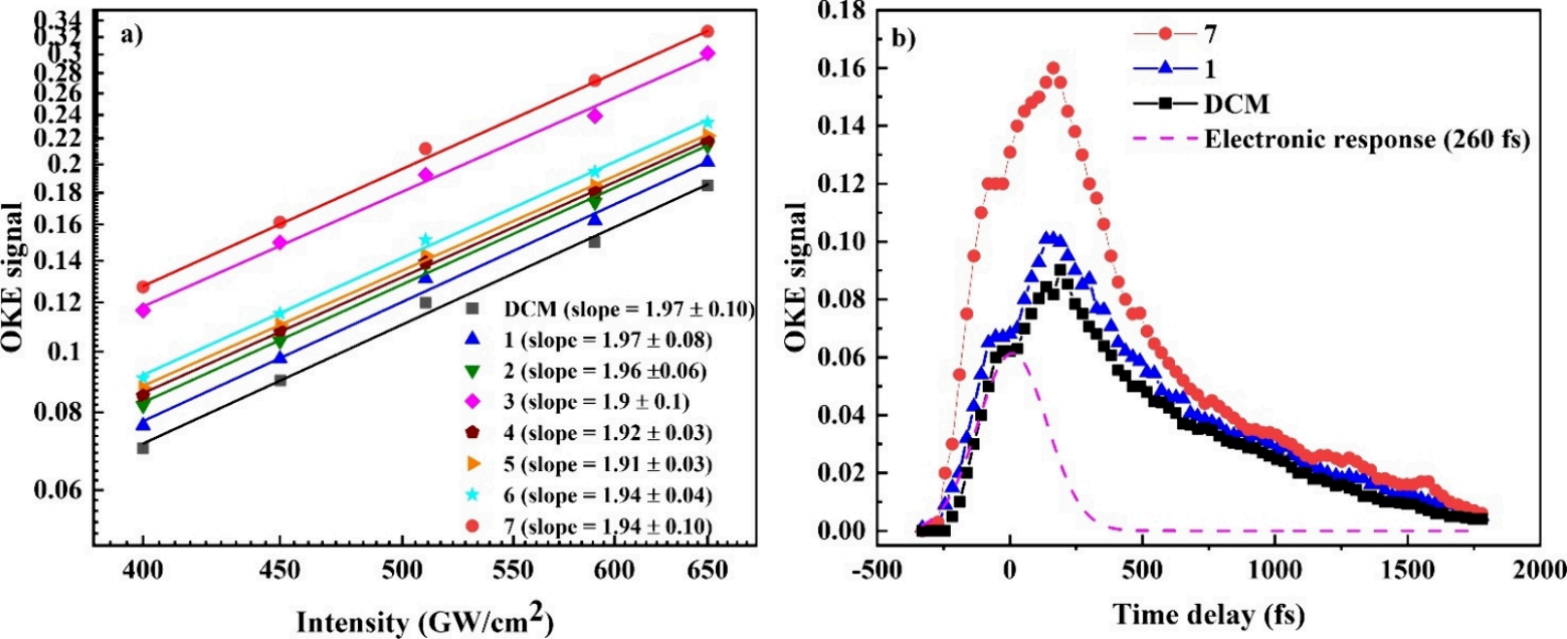
(a) Dependence of the OKE signals of compounds 1–7 and DCM and (b) time evolution of the OKE signal of compounds 1 and 7 and DCM (all recordings correspond to a pump intensity of 450 GW/cm^2^).

**Figure 10:**
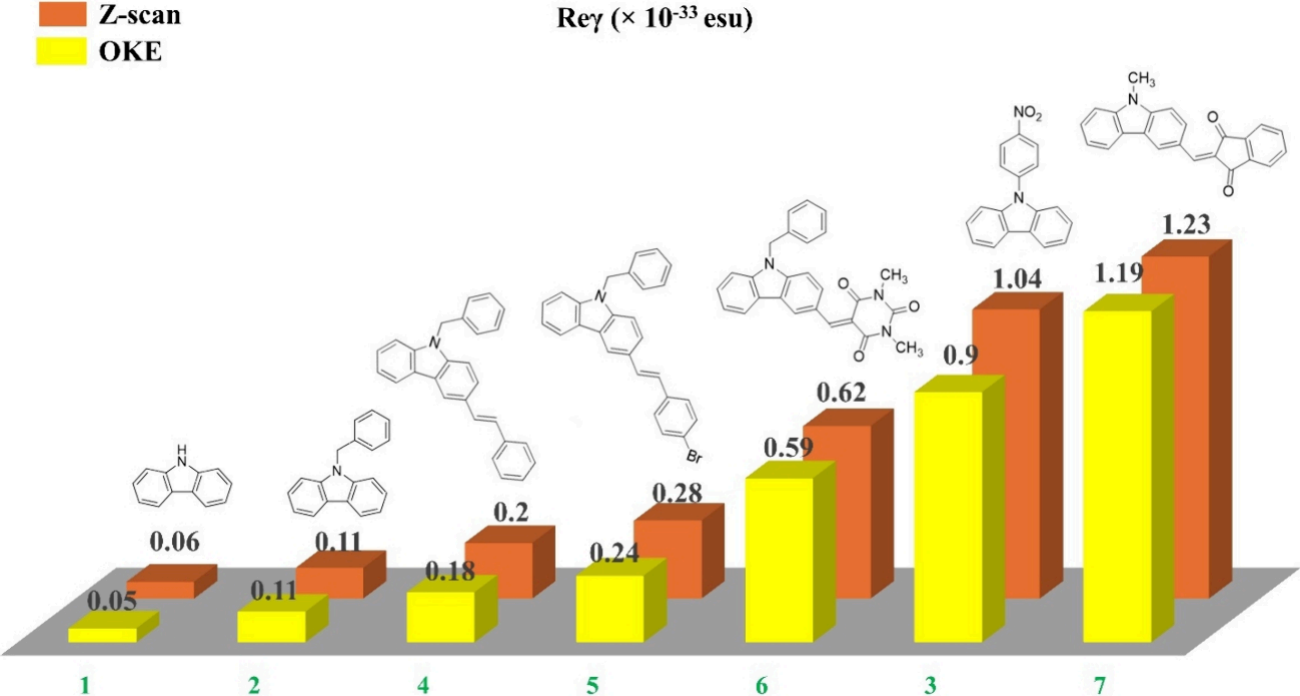
Comparison of the values of the real part of second-order hyperpolarizability (Reγ) of compounds 1–7 obtained by *Z*-scan and OKE techniques.

The results of investigating the NLO response of the studied molecules by using the OKE technique are presented in Figure 9. First, the dependence of the OKE signal on the pump beam intensity for the compounds 1–7 as well as DCM was investigated for the assessment of their NLO refractive response (Figure 9a). These measurements were performed with the intensity of probe beam set constant at ∼50 GW/cm^2^, whereas the pump beam was varied. In all cases, the OKE signals displayed quadratic dependence on the pump laser intensity, indicative of third-order optical nonlinearities.^65^ Utilizing eqs 5 and 8 and DCM as the reference material, with a Reχ^(3)^ value of about (27.8 ± 1.0) × 10^–16^ esu, determined in this work through *Z*-scan measurements, the Reγ values of the studied carbazole-based dyes were derived and are presented in Figure 10. As evident from this figure, there is excellent consistency of the Reγ values obtained by the two techniques. Furthermore, given that the OKE measurements confirmed the absence of higher-order nonlinearities, the NLO absorption observed in the studies dyes can be ascribed to TPA. It should be highlighted that, to the best of our knowledge, this is the first time the OKE technique has been used to determine the order of the multiphoton process in dyes promising as initiators for MPL.

In addition, to gain a better understanding of the mechanisms contributing to the NLO response of the studied dyes, time-resolved OKE measurements were also conducted. In Figure 9b, recordings providing the temporal evolution of the OKE signal for solutions of compounds 1 and 7, along with neat solvent, are shown as an example. The first peak (on the left side) observed at zero-time delay signifies the instantaneous electronic response contribution to the OKE signal. Fitting this peak with a Gaussian-type function provides the autocorrelation profile of the laser, which showed a pulse width of approximately 260 fs. The adjacent, more intense peak corresponds to the contribution of molecular reorientation on the NLO response, which is followed by an exponential-like decay of the molecular system as it relaxes toward the equilibrium.^72,73^

**Figure 11:**
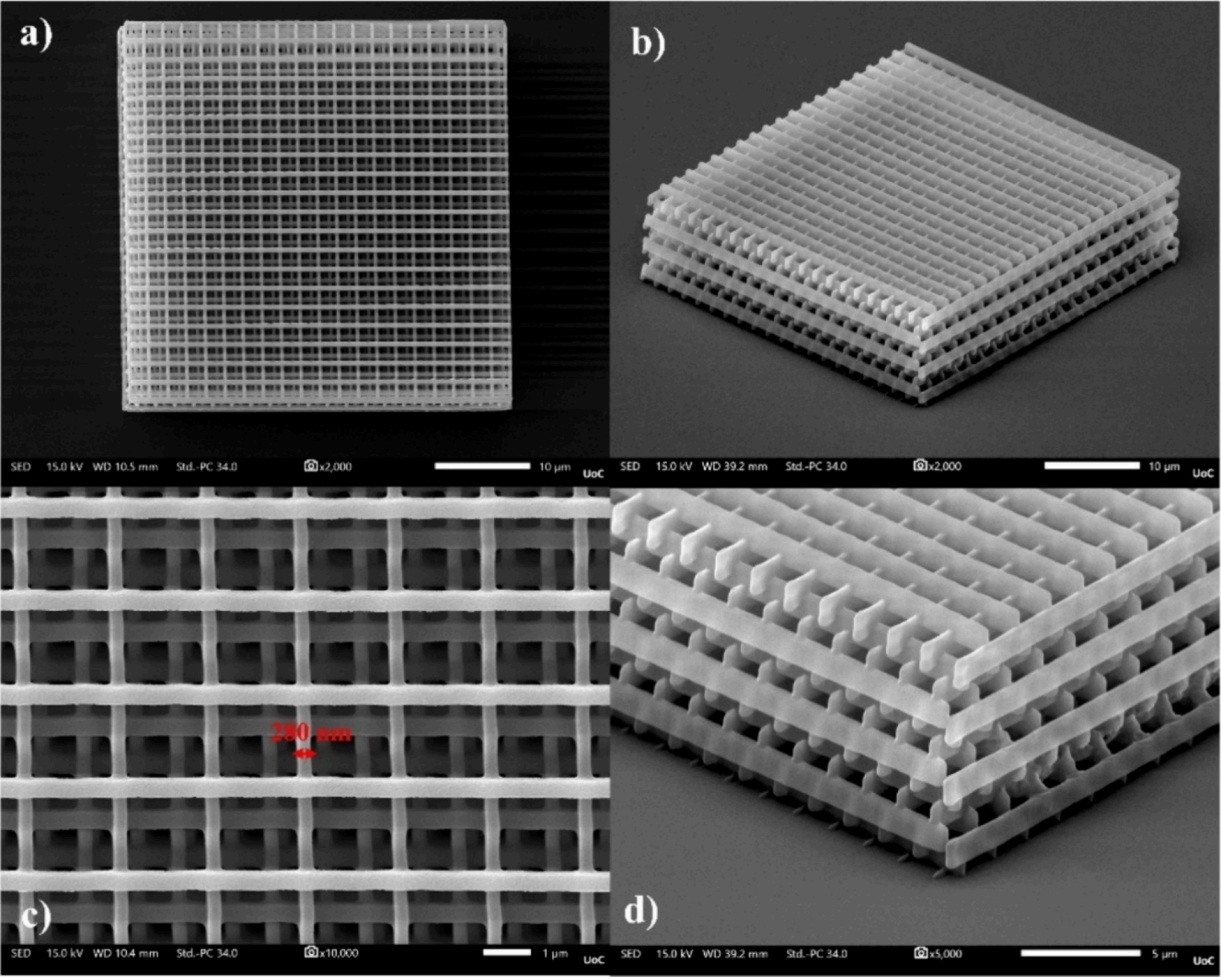
SEM images of a woodpile structure fabricated using compound 7 as photoinitiator: (a) top view, (b) tilted view, and (c, d) structure detail.

To assess the effectiveness of carbazole-based dyes as PIs for MPL and to evaluate the impact of carbazole’s chemical functionalization on critical aspects of the printing process, such as feature size and laser intensity to achieve optimal fabrication, structuring tests were conducted using compounds 1 and 7. For this purpose, the ideal building parameters for each compound were determined by varying the laser intensities and stage velocity. The first 3D structure fabricated was a woodpile structure composed of layers of one-dimensional rods stacked in a repeating pattern, typically with a periodicity of every four layers. In the context of MPL, this structure is commonly employed as test pattern for assessing the material’s structurability and resolution, enabling comparisons with other studies.^46−48,50,74^ In Figure 11, an example of such a 3D-structure fabricated using the zirconium–silicon hybrid is shown, with 1% w/w of the compound 7 used as PI. This compound was selected among the others for MPL experiments because it exhibited a superior NLO absorptive response, which is crucial for achieving high-resolution and efficient fabrication.^75^ As presented, the fabricated woodpile structure demonstrated a low shrinkage and achieved a low feature size of ∼280 nm. The laser intensity needed for fabricating high-quality structures without presenting any deformations was ∼1.14 TW/cm^2^, measured before the objective lens, and the writing speed was 50 μm/s. As shown in Figure S3, the obtained feature size is comparable to that achieved with other widely used initiators in MPL applications, such as 4,4′-bis(diethylamino)benzophenone,^50^ Irgacure derivatives,^74^ acylophosphine oxides,^48^ ketones,^47^ and thioxanthones.^46^

On the other hand, as can be seen in Figure S4, when using unfunctionalized carbazole (i.e., compound 1) as the radical initiator, the achievable feature size was significantly larger, approximately ∼416 nm. This can be ascribed to the lesser extent of electron delocalization in the p orbitals of the carbazole molecule compared to compound 7. Furthermore, it should be emphasized that when setting the writing speed at 50 μm/s, the corresponding ideal building laser intensity was approximately 3.1 TW/cm^2^, which is three times larger compared to that required for fabricating woodpile structures with compound 7. Trials using compound 7 as a PI, with the laser intensity set at 3.1 TW/cm^2^, resulted in burning of the structure. These findings undoubtedly indicate the effectiveness of the chemical functionalization of carbazole in enhancing resolution and MPL performance.

**Figure 12:**
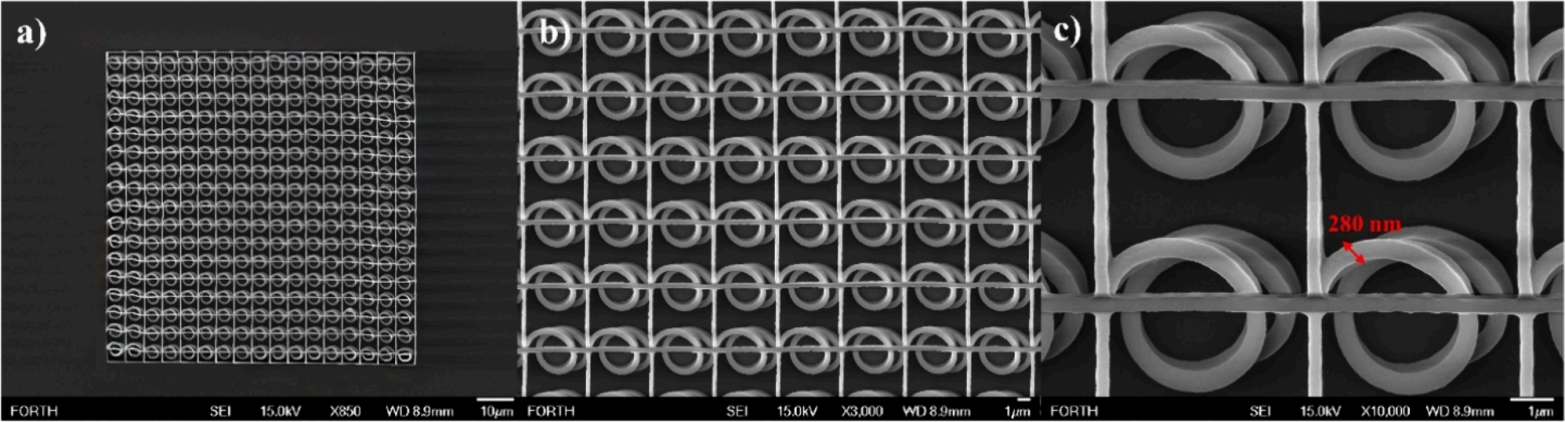
SEM images of a circular spiral architecture structure fabricated using compound 7 as photoinitiator: (a) top view and (b, c) structure detail.

To emphasize the versatility of the studied carbazole-based organic materials as a novel class of photoinitiators for MPL applications, a spiral photonic crystal was also fabricated by using compound 7 under 515 nm laser radiation. Spiral photonic crystals can be designed to trap or guide light and are thus of considerable interest for use in optics and communications.^76^ Representative SEM images of circular spiral architecture structures demonstrating effective fabrication using compound 7 are illustrated in Figure 12. The optimal printing parameters, i.e., laser intensity and scanning speed, for this structure were determined to be approximately 1.1 TW/cm^2^ and 50 μm/s, respectively.

The obtained findings indicate that carbazole-based organic materials are effective for MPL applications by using visible laser light at 515 nm. This is attributed to the multiple aspects of chemical functionalization of the carbazole moiety, including N atoms and aromatic rings, enabling the generation of various D–A and D−π–Α architectures with wide energy band gaps. Additionally, these architectures, featuring strong electron-donating and -accepting groups, can lead to high spatial resolutions, effective MPL performance, and reduced photodamage, likely due to efficient charge transfer.

## Conclusions

In summary, the present work investigated the ultrafast NLO response of some carbazole-based organic materials with D−π–Α and D–A architectures with the aim to explore their potential in optoelectronics and photonics. The studied compounds, which exhibit different push–pull electron abilities, were found to exhibit strong NLO properties, which were significantly heightened compared with pristine carbazole. This enhancement was translated into lower feature size microstructures fabricated via MPL and the ability to achieve optimal fabrication utilizing lower laser intensities, induced by electron delocalization extension and charge transfer effects. The findings of this study highlight the importance of chemical functionalization in improving and tailoring the NLO response of carbazole. By expanding the understanding of carbazole-based compounds’ NLO characteristics, this research opens up new avenues for innovation and application in various optoelectronic and photonic devices.

## Supporting Information Available

Nonlinear optical properties of DCM; schematic presentation of the electronic transitions in compounds 2 and 3; woodpile structure fabricated using compound 1 as photoinitiator; NLO properties of compounds 1–7 under 246 fs, 515 nm laser excitation; feature size and fabrication speed of structures using other photoinitiators; and NLO absorption properties of other photoinitiators (PDF)
