# Supplementary material for: Push–Pull Carbazole-Based Dyes: Synthesis, Strong Ultrafast Nonlinear Optical Response, and Effective Photoinitiation for Multiphoton Lithography
Source: ACS Appl Opt Mater. 2024 Aug 5;2(8):1653–66. doi: 10.1021/acsaom.4c00241 (PMC11348418; doi:10.1021/acsaom.4c00241)
Supplement: Supplementary file 1 — ot4c00241_si_001.pdf [file ot4c00241_si_001.pdf]

Supporting Information

Push-Pull Carbazole-Based Dyes: Synthesis, Strong Ultrafast Nonlinear Optical Response, and Effective Photoinitiation for Multiphoton Lithography

Michalis Stavrou,[1] Gordon Zyla,[1] Dimitra Ladika,[1] Frederic Dumur,[2] Maria Farsari,[1] David Gray[1*]*

[1]Institute of Electronic Structure and Laser, Foundation for Research and Technology-Hellas, 70013 Heraklion, Greece

[2] Aix Marseille Univ, CNRS, ICR, UMR 7273, F-13397 Marseille, France

Corresponding Author

* Michalis Stavrou (email: m.stavrou@iesl.forth.gr)

* David Gray (email: dgray@iesl.forth.gr)

Nonlinear optical properties of DCM

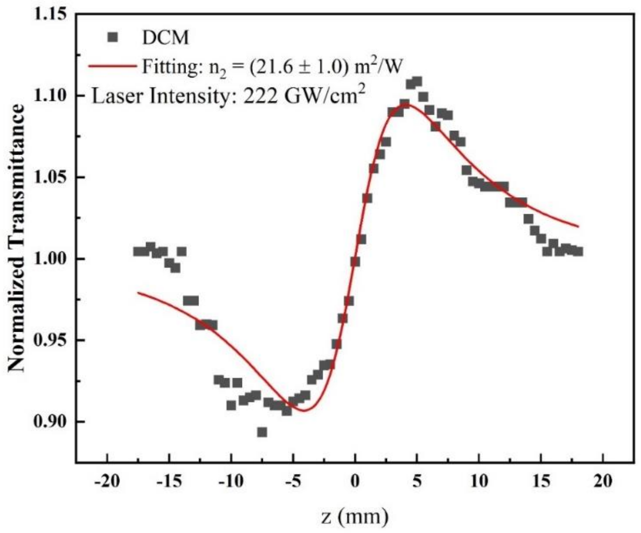

Figure S1. CA Z-scan recording of DCM.

Electronic transitions in compounds 2 and 3

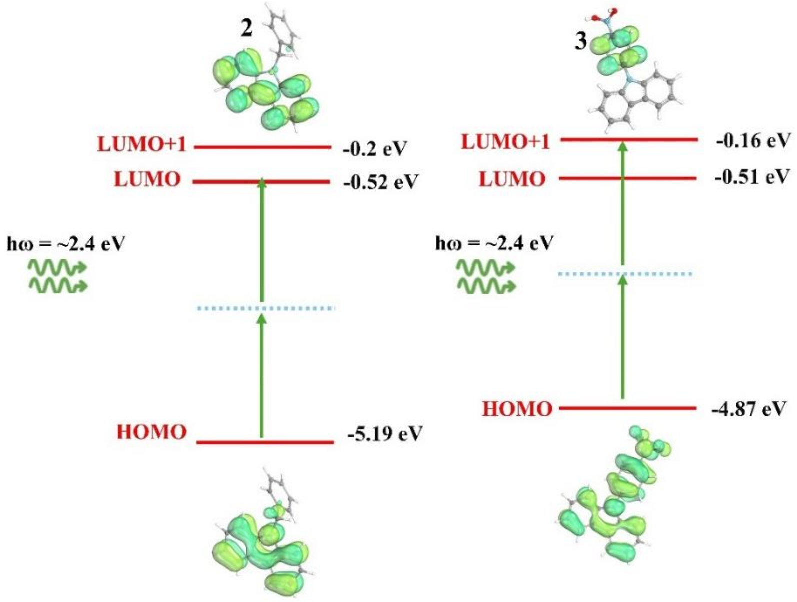

Figure S2. Description of the electronic transitions occurring in compounds 2 and 3 for fs laser excitation at 515 nm.

Comparison of the structures' feature size and fabrication speed with those of structures, fabricated using other initiators

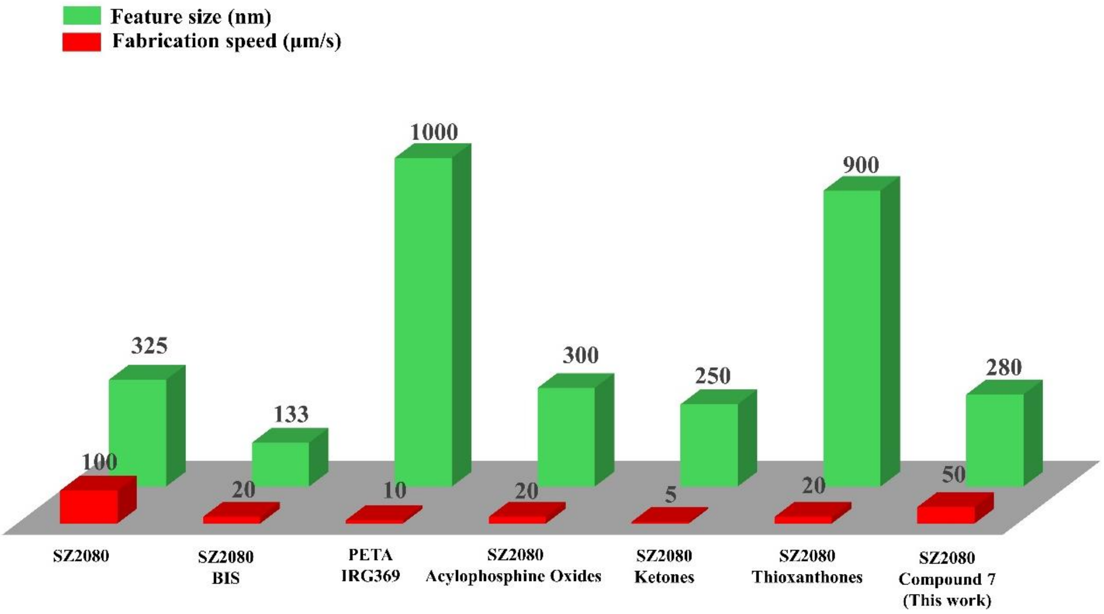

Figure S3. Comparison of the feature size and fabrication speed of microstructures fabricated using SZ2080 with compound 7 with those fabricated using SZ2080 without a photoinitiator[1] and with widely used photoinitiators, such as 4,4'-bis(diethylamino)benzophenone (BIS),[2] acylophosphine oxides,[3] ketones,[4] and thioxanthones,[5] as well as PETA with Irgacure (IRG) 369.[6]

Woodpile structure fabricated using compound 1 as photoinitiator

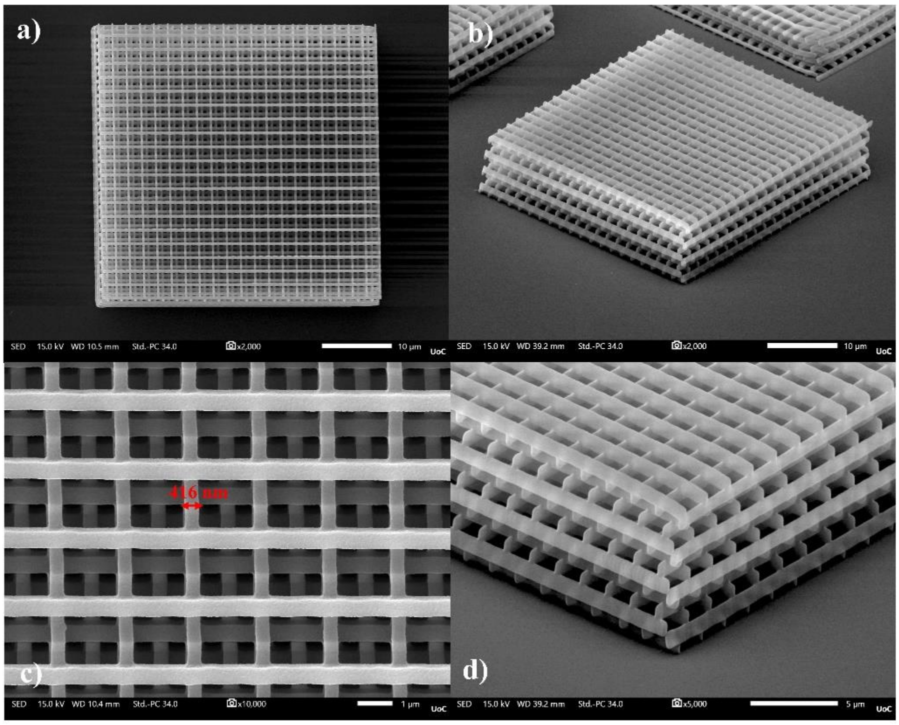

Figure S4. SEM images of a woodpile structure fabricated using compound 1 as photoinitiator: (a) top view, (b) tilted view and (c, d) structure detail.

NLO properties of the compounds (1-7) and comparisons with other efficient photoinitiators

Table S1. NLO properties (β: nonlinear absorption coefficient, n_2: nonlinear refractive index, $Im\chi^{(3)}$: imaginary part of the third-order susceptibility, $Re\chi^{(3)}$: real part of the third-order susceptibility $Im\gamma$: imaginary part of second-order hyperpolarizability, $Re\gamma$: real part of second-order hyperpolarizability, γ: second-order hyperpolarizability and σ: two-photon absorption cross section) of compounds (1-7) after subtracting solvent's contribution, under 246 fs, 515 nm laser excitation.

Compound	C (mM)	β (x 10^{-15} m/W)	n_2 (x 10^{-21} m^2/W)	$Im\chi^{(3)}$ (x 10^{-16} esu)	$Re\chi^{(3)}$ (x 10^{-16} esu)	Imγ (x 10^{-33} esu)	Reγ (x 10^{-33} esu)	γ (x 10^{-33} esu)	σ (GM)
1	1.1	30.6 ± 2.0	1.1 ± 0.1	9.7 ± 0.6	1.4 ± 0.1	0.37 ± 0.02	0.06 ± 0.01	0.37 ± 0.02	0.18 ± 0.01
2	0.78	51.9 ± 2.0	1.7 ± 0.2	16.4 ± 0.6	2.2 ± 0.3	0.83 ± 0.03	0.11 ± 0.10	0.84 ± 0.09	0.43 ± 0.02
3	0.35	103.8 ± 4.0	-7.1 ± 0.6	33.4 ± 1.0	-9.1 ± 0.8	3.8 ± 0.1	-1.04 ± 0.09	4.0 ± 0.1	2.0 ± 0.1
4	0.56	54.1 ± 2.0	2.2 ± 0.1	17.5 ± 0.6	2.8 ± 0.1	1.2 ± 0.1	0.20 ± 0.01	1.21 ± 0.10	0.59 ± 0.02
5	0.46	50.1 ± 2.0	2.6 ± 0.2	16.2 ± 0.6	3.3 ± 0.3	1.4 ± 0.1	0.28 ± 0.02	1.43 ± 0.10	0.70 ± 0.03
6	0.24	63.9 ± 3.0	2.9 ± 0.2	20.2 ± 1.0	3.7 ± 0.3	3.36 ± 0.20	0.62 ± 0.05	3.42 ± 0.02	1.71 ± 0.08
7	0.3	111 ± 8	7.2 ± 0.5	36 ± 2	9.3 ± 0.6	4.7 ± 0.3	1.23 ± 0.08	4.9 ± 0.3	2.37 ± 0.20

Table S2. NLO absorption-related parameters properties of 4,4'-bis(diethylamino)benzophenone, Irgacure series (e.g., 184, 369, 754, 819 and 907 series), thionxanthones (e.g., 1-chloro-4-propoxythioxanthone and bromo-substituded thionxathones), ketones (e.g., indene-1,3-dione functionalized ketone), and acyl-ophosphine oxide.

Compound	Excitation conditions	C (mM)	Imγ (x 10^{-33} esu)	σ (GM)
4,4'-bis(diethylamino)benzophenone	800 nm, 60 fs	250	13.6	7
Irgacure 907	775 nm, 150 fs	10	5.4	4
Irgacure 819		10	< 5.4	< 4
Irgacure 754		10	28.2	21
Irgacure 369		10	9.4	7
Irgacure 184		10	30.9	23
1-chloro-4-propoxythioxanthone	800 nm, 146 fs	16	13.3	7
Bromo-substituted thioxanthones	800 nm, 60 fs	50	5-11.2	4-9
indene-1,3-dione functionalized ketone	1064 nm, 90 ps	n/a	0.3	0.22
acyl−phosphine oxide	800 nm, 250 fs	150	13.7	9

REFERENCES

(1) Butkus, A.; Skliutas, E.; Gailevičius, D.; Malinauskas, M. Femtosecond-Laser Direct Writing 3D Micro/Nano-Lithography Using VIS-Light Oscillator. *J. Cent. South Univ.* **2022**, *29*, 3270–3276.

(2) Sakellari, I.; Kabouraki, E.; Gray, D.; Purlys, V.; Fotakis, C.; Pikulin, A.; Bityurin, N.; Vamvakaki, M.; Farsari, M. Diffusion-Assisted High-Resolution Direct Femtosecond Laser Writing. *ACS Nano* **2012**, *6*, 2302–2311.

(3) Im, J.; Liu, Y.; Hu, Q.; Trindade, G. F.; Parmenter, C.; Fay, M.; He, Y.; Irvine, D. J.; Tuck, C.; Wildman, R. D.; *et al.* Strategies for Integrating Metal Nanoparticles with Two-photon Polymerization Process: Toward High Resolution Functional Additive Manufacturing. *Adv. Funct. Mater.* **2023**, *33*, 2211920.

(4) Nazir, R.; Danilevicius, P.; Gray, D.; Farsari, M.; Gryko, D. T. Push–Pull Acylo-Phosphine Oxides for Two-Photon-Induced Polymerization. *Macromolecules* **2013**, *46*, 7239–7244.

(5) Perevoznik, D.; Nazir, R.; Kiyan, R.; Kurselis, K.; Koszarna, B.; Gryko, D. T.; Chichkov, B. N. High-Speed Two-Photon Polymerization 3D Printing with a Microchip Laser at its Fundamental Wavelength. *Opt. Express* **2019**, *27*, 25119-251225.

(6) Nazir, R.; Balčiūnas, E.; Buczyńska, D.; Bourquard, F.; Kowalska, D.; Gray, D.; Maćkowski, S.; Farsari, M.; Gryko, D. T. Donor–Acceptor Type Thioxanthones: Synthesis, Optical Properties, and Two-Photon Induced Polymerization. *Macromolecules* **2015**, *48*, 2466–2472.
